# Supplementary material for: Kinesin-1 mediates proper ER folding of the CaV1.2 channel and maintains mouse glucose homeostasis
Source: EMBO Rep. 2024 Sep 25;25(11):11. doi: 10.1038/s44319-024-00246-y (PMC11549326; doi:10.1038/s44319-024-00246-y)
Supplement: Supplementary file 2 — Movie EV1 [file 44319_2024_246_MOESM2_ESM.zip › Movie EV1 readme.docx]

**Movie EV1. Exocytosis of a glucose-stimulated beta cells**

Time-lapse observations of insulin granule exocytosis in synapto.pHluorin-transduced primary beta cells of the indicated genotypes with a TIRF microscope. The whole movie corresponds to 1 min. Corresponding to Fig. 2A.
