# Supplementary material for: Kinesin-1 mediates proper ER folding of the CaV1.2 channel and maintains mouse glucose homeostasis
Source: EMBO Rep. 2024 Sep 25;25(11):11. doi: 10.1038/s44319-024-00246-y (PMC11549326; doi:10.1038/s44319-024-00246-y)
Supplement: Supplementary file 3 — Movie EV2 [file 44319_2024_246_MOESM3_ESM.zip › Movie EV2 readme.docx]

**Movie EV2. Mobility of insulin granules in primary beta cells.**

The mobility of insulin granules tagged with EGFP-phogrin in control primary beta cells, followed by that in cKO primary beta cells. The time lapse covers a period of 100 s approximately 30 min after glucose stimulation. Note that the mobility of insulin granules in cKO cells is decreased compared with that in control cells. Corresponding to Fig. 3C–H.
