# Supplementary material for: Kinesin-1 mediates proper ER folding of the CaV1.2 channel and maintains mouse glucose homeostasis
Source: EMBO Rep. 2024 Sep 25;25(11):11. doi: 10.1038/s44319-024-00246-y (PMC11549326; doi:10.1038/s44319-024-00246-y)
Supplement: Supplementary file 4 — Movie EV3 [file 44319_2024_246_MOESM4_ESM.zip › Movie EV3 readme.docx]

Movie EV3. KIF5B-dependent dynamics of peripheral F-actin in beta cells.

F-actin dynamics of control (CT) and cKO (KO) beta cells labeled by a *Lifeact-mCherry* transgene upon glucose stimulation. Scale bar, 5 μm. The duration of the movie corresponds to 28 min. Corresponding to Figs. 4A and EV2A.
