# Supplementary material for: Kinesin-1 mediates proper ER folding of the CaV1.2 channel and maintains mouse glucose homeostasis
Source: EMBO Rep. 2024 Sep 25;25(11):11. doi: 10.1038/s44319-024-00246-y (PMC11549326; doi:10.1038/s44319-024-00246-y)
Supplement: Supplementary file 5 — Movie EV4 [file 44319_2024_246_MOESM5_ESM.zip › Movie EV4 readme.docx]

Movie EV4. KIF5B is largely dispensable for post-Golgi trafficking of the Ca_V_1.2 protein

A BFA washout assay of Ca_V_1.2-EGFP in primary beta cells of the indicated genotypes. Bar, 5 μm. The Movie shows from 15 min to 119 min after the BFA washout. Corresponding to Fig. 6D.
