# Supplementary material for: Kinesin-1 mediates proper ER folding of the CaV1.2 channel and maintains mouse glucose homeostasis
Source: EMBO Rep. 2024 Sep 25;25(11):11. doi: 10.1038/s44319-024-00246-y (PMC11549326; doi:10.1038/s44319-024-00246-y)
Supplement: Supplementary file 6 — Movie EV5 [file 44319_2024_246_MOESM6_ESM.zip › Movie EV5 readme.docx]

**Movie EV5. Dynamics of cell bottom microdroplets containing Hsp90 and KIF5B**

Time-lapse recording in the bottom region of a primary wild-type mouse beta cell expressing tagRFP-Hsp90 (red) and KIF5B-EYFP (green). Scale bar, 1 μm. The duration of the movie corresponds to 464 s. Corresponding to Fig. 8A.
