# Supplementary material for: Kinesin-1 mediates proper ER folding of the CaV1.2 channel and maintains mouse glucose homeostasis
Source: EMBO Rep. 2024 Sep 25;25(11):11. doi: 10.1038/s44319-024-00246-y (PMC11549326; doi:10.1038/s44319-024-00246-y)
Supplement: Supplementary file 7 — Movie EV6 [file 44319_2024_246_MOESM7_ESM.zip › Movie EV6 readme.docx]

**Movie EV6. Dynamics of Hsp90 in an ER sheet**

Multicolor time-lapse recording in the bottom region of a wild-type primary mouse beta cell, expressing tagRFP-Hsp90 (red) and mEmerald-Sec61β (green). Scale bar, 1 μm. The movie covers a period of 76 s. Corresponding to Fig. 8G.
