# Supplementary material for: Kinesin-1 mediates proper ER folding of the CaV1.2 channel and maintains mouse glucose homeostasis
Source: EMBO Rep. 2024 Sep 25;25(11):11. doi: 10.1038/s44319-024-00246-y (PMC11549326; doi:10.1038/s44319-024-00246-y)
Supplement: Supplementary file 8 — Expanded View Figures [file 44319_2024_246_MOESM8_ESM.pdf]

Expanded View Figures

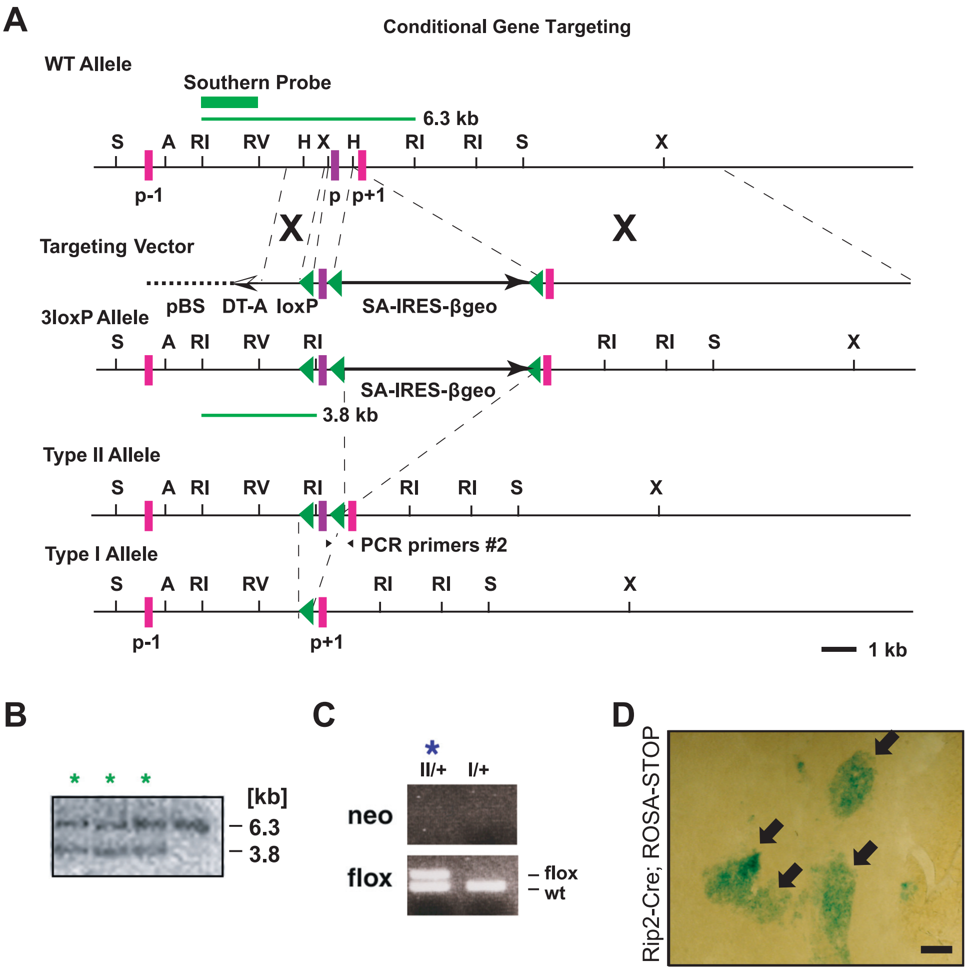

Figure EV1. Conditional knockout of mouse *Kif5b* gene.

(**A–D**) Establishment of beta-cell-specific *Kif5b* gene conditional knockout (cKO) mice, represented by a gene targeting strategy in mouse ES cells (**A**), Southern blotting screening for homologous recombinants (**B**; asterisks); genotyping PCR for the floxed allele (**C**: asterisk); and characterization of Rip2-Cre activity in a pancreas section detected by a LacZ reporter, ROSA-STOP mice (**D**). p, the 74 bp P-loop exon flanked by *loxP* sites (green triangles). S, *SalI*; A, *ApaI*; RI, *EcoRI*; RV, *EcoRV*; H, *HindIII*; X, *XbaI*. Arrows in (**D**), specific Cre/*loxP* recombination sites in the pancreas of a *Rip2-Cre ROSA-STOP* double heterozygous mouse. Scale bar, 100 µm. Corresponding to Fig. 1A–C.

© The Author(s)

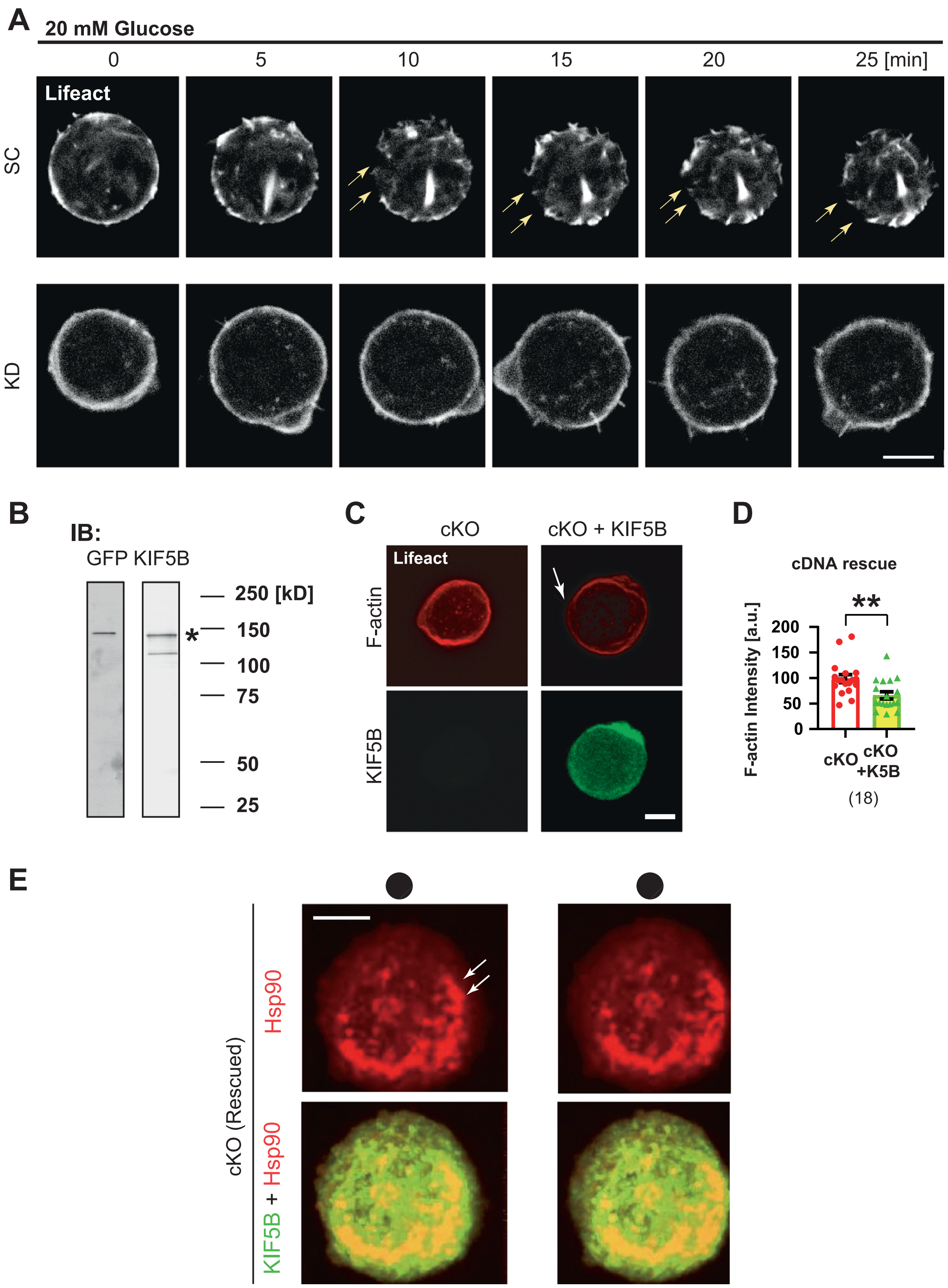

Figure EV2. KIF5B facilitates cortical actin remodeling.

(**A**) Time-lapse study of glucose-stimulated actin remodeling of primary beta cells from *Lifeact-mCherry* transgenic mouse pancreas, transduced with scrambled-control (SC) or KIF5B-knockdown (KD) miRNA expression vectors. Scale bar, 5 μm. Arrows, actin remodeling. Corresponding to Fig. 4A and Movie EV3. (**B–D**) Rescue study of the glucose-stimulated actin remodeling in cKO primary beta cells by transducing KIF5B-EYFP, represented by immunoblotting of the expressed proteins in Ins1 cells using a mouse anti-GFP antibody and a rabbit anti-KIF5B antibody (**B**), *Lifeact-mCherry* transgene labeling (**C**), and F-actin quantification (**D**). Scale bar, 5 μm. Asterisks in (**B**), bands for tagged KIF5B. Arrow in (**C**), actin remodeling. $^{**}P = 0.0042$, two-sided unpaired Welch's *t* test; $n = 18$. Corresponding to Fig. 4A. (**E**) Stereoscopic fluorescence microscopy of a cKO primary beta-cell expressing tagRFP-Hsp90 (red) and KIF5B-EYFP (green). Scale bar, 5 μm. Data are represented by the mean ± SEM. Corresponding to Fig. 8A.

 © The Author(s)
